# Transumbilical Multiport Laparoscopic Nephroureterectomy for Congenital Renal Dysplasia in Children: Midterm Follow-Up from a Single Institution

**DOI:** 10.3389/fped.2013.00046

**Published:** 2013-12-13

**Authors:** Hong Mei, Teng Qi, Shuai Li, Jiarui Pu, Guoqing Cao, Shaotao Tang, Liduan Zheng, Qiangsong Tong

**Affiliations:** ^1^Department of Pediatric Surgery, Union Hospital of Tongji Medical College, Huazhong University of Science and Technology, Wuhan, Hubei Province, China; ^2^Department of Pathology, Union Hospital of Tongji Medical College, Huazhong University of Science and Technology, Wuhan, Hubei Province, China; ^3^Clinical Center of Human Genomic Research, Union Hospital of Tongji Medical College, Huazhong University of Science and Technology, Wuhan, Hubei Province, China

**Keywords:** transumbilical multiport, laparoscopic nephroureterectomy, renal dysplasia, children, follow-up

## Abstract

**Objective:** To assess the clinical utility and efficiency of transumbilical multiport laparoscopic nephroureterectomy (TMLN) for the treatment of congenital renal dysplasia in children by analyzing consecutive cases from a single institution.

**Methods:** Sixteen children underwent TMLN procedure due to dysplastic kidney between January 2010 and December 2011. The surgery was transperitoneally performed through three transumbilical incisions for two 5-mm and one 3-mm ports, which duplicated the standard laparoscopic steps with the usual laparoscopic instruments. Demographic, perioperative, and follow-up data were analyzed.

**Results:** TMLN was performed in all patients, without additional ports or conversion to open surgery. The mean operation time was 108.4 min (range 90–125), and the blood loss was minimal. There were no severe intraoperative or post-operative complications. The post-operative recovery was uneventful in all patients. No urinary incontinence or umbilical hernias occurred. The cosmetic result was excellent as the incision scar was hidden inside the belly button.

**Conclusion:** TMLN is a safe and efficient procedure for the management of congenital renal dysplasia in children with good cosmesis. Future randomized studies with a larger number of cases and a longer follow-up are warranted to elucidate the benefits and limitations of TMLN in children.

## Introduction

Congenital renal dysplasia is a consequence of abnormal nephrogenesis, and histologically characterized by dysplastic nephrons and collecting ducts (1). The natural history of congenital renal dysplasia is associated with gradual deterioration of kidney function, urinary tract infection, and hypertension in the pediatric population (1). As the final consequence of abnormal nephrogenesis, renal dysplasia is usually diagnosed based on a combination of clinical and radiological findings (2). In recent years, laparoscopic nephroureterectomy has evolved into the accepted procedure for the treatment of dysplastic kidney in pediatric patients (3). Conventional laparoscopic approaches usually require three to four incisions for trocar placement, and each additional port may result in undesirable cosmetic outcome and potential incision complications, such as bleeding from abdominal wall vessels, internal organ damage (bowel injury), incisional hernia, and wound infections (4, 5). With the development of natural-orifice transluminal endoscopic surgery (NOTES), laparoendoscopic single-site surgery (LESS) has become the focus of minimally invasive urology to minimize the number of incisions and decrease morbidity (5). After the first description of laparoscopic single-port-access nephrectomy in a pediatric patient with a multicystic dysplastic kidney by Johnson et al. in 2009 (4), this approach has recently been applied for congenital renal dysplasia in children. Despite the widespread acceptance of standard LESS, technical challenges and higher costs for more sophisticated instruments limit the generalization of LESS in many medical centers, especially in the developing countries. In this study, based on the principles of LESS and triangulation of laparoscopy, we described the feasibility and efficiency of an alternative procedure, named as transumbilical multiport laparoscopic nephroureterectomy (TMLN) in pediatric patients, which applied three transumbilical ports and usual laparoscopic instruments for the treatment of renal dysplasia.

## Patients and Methods

### Patients

To assess the clinical utility and efficiency of TMLN, with the institutional review board approval, we respectively reviewed 16 consecutive pediatric patients with dysplastic kidney, who underwent TMLN between January 2010 and December 2011 at the Department of Pediatric Surgery, Union Hospital of Tongji Medical College, China. Six patients suffered from urinary incontinence due to the ectopic ureter. The demographic characteristics of all patients were summarized in Table 1. The preoperative ultrasonography and magnetic resonance urography of these patients indicated a small dysplastic kidney with or without an ipsilateral ectopic ureter. Renal scintigraphy revealed a faintly observed dysplastic kidney with <1% function in the dysplastic kidney, and a normal contralateral kidney. The indication for TMLN referred to a dysplastic kidney with or without an ectopic ureter. TMLN procedures were performed by the same experienced surgeon (Tong Q) with the same surgical team.

**Table 1:** **Demographics details of patients**.

Characteristic	Transumbilical multiport laparoscopic nephroureterectomy
No. of cases	16
Age (months)	33.8 (range: 7–62)
Gender (M/F)	5/11
Side (L/R)	12/4
Urinary incontinence	6
Ectopic ureter	6

*M, male; F, female; L, left; R, right*.

### Surgical procedures

**Figure 1:**
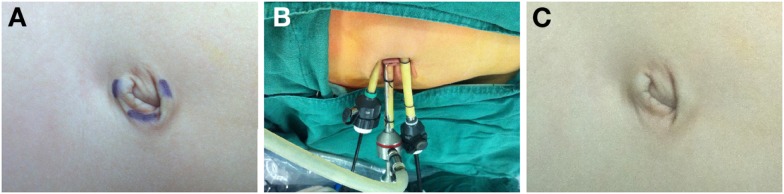
**Placement of transumbilical ports for TMLN and post-operative appearance**. **(A)** Preoperative design of transumbilical port position. **(B)** One 5-mm port at the contralateral aspect and 9 o’clock position of umbilicus for the camera, and two additional 5-mm and 3-mm working ports inserted through the periumbilical skin incision at the 6 and 12 o’clock position, respectively. **(C)** Umbilical appearance 4 weeks after the surgery.

After the induction of general anesthesia, the patients were secured to the operating room table, and placed in the modified lateral decubitus position. A Foley urethral catheter was positioned to decompress the bladder. The oral-gastric tube was not routinely applied. Three ports were transumbilically placed at different positions and staggered in high, middle, and low dimensions (Figure 1A), including one 5-mm port for the camera at the contralateral aspect and 9 o’clock position of umbilicus for left nephrectomy or 3 o’clock position for right nephrectomy, and one 5-mm and one 3-mm additional working ports inserted through the periumbilical skin incision at the 6 and 12 o’clock position, respectively. To avoid the instrument collision and enlarge the operating space, during the insertion of two working ports, the trocars were laterally placed along subcutaneous planes before penetrating the peritoneum, resulting in longer intra-abdominal distance between the two instruments. The assistant was standing next to the surgeon, keeping the camera usually at a position that was above or at the middle of two working instruments. The instruments used in TMLN were similar to those in standard laparoscopic nephroureterectomy, and the 3-mm working instrument was always a grasper for retraction, while the other was the main dissecting instrument (Figure 1B).

**Figure 2:**
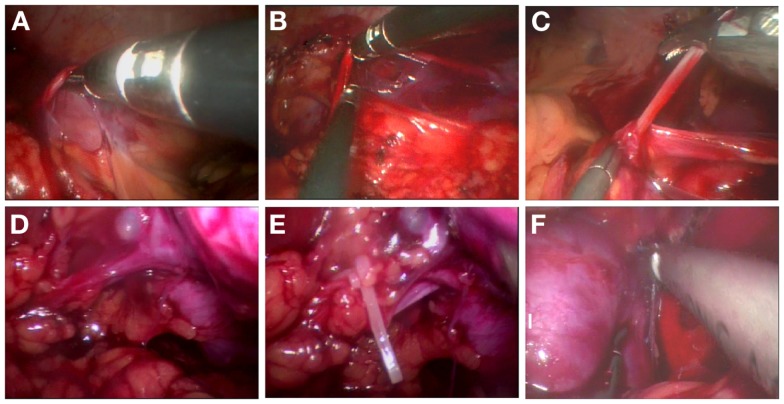
**Surgical procedure of TMLN for the treatment of a left-side dysplastic kidney**. **(A)** Identification of the ureter as it crosses the iliac vessels in the pelvis. **(B)** Dissociation of the ureter to the lower pole level of kidney. **(C)** Pulling up of the ureter to provide good access to the renal pedicle. **(D)** Mobilization and identification of the renal pedicle. **(E)** Ligation of renal vessels by the 5-mm Hem-o-lok. **(F)** Further mobilization and dissection of the dysplastic kidney.

Pneumoperitoneum was created through the primary 5-mm port using carbon dioxide to a maximum pressure of 10–12 mmHg, and was maintained at 8 mmHg during surgery. A rigid, 5-mm, 30° telescope was introduced into the abdomen for an initial survey. The ureter was identified as it crossed the iliac vessels in the pelvis (Figure 2A), and was dissected cephalad to the lower pole level of kidney (Figure 2B). The ureter was pulled up to provide good access to the renal pedicle and improve exposure of the tissue planes (Figure 2C). After sparing mobilization of the colon and identification of the affected kidney, the renal pedicle was mobilized and identified (Figure 2D). The small renal vessels supplying the dysplastic kidney were either clipped using the 5-mm Hem-o-lok or coagulated, and then divided with a Harmonic scalpel (Ethicon, Cincinnati, OH, USA) (Figure 2E). Once the renal vessels were divided, the periphery of Gerota’s fascia was further dissected until the kidney was completely mobilized (Figure 2F). The ureter was completely isolated as low as possible without damage of the ovarian vein or vas deferens. In some cases, a transabdominal hitch stitch was placed through the ureter and allowed the distal ureterectomy near the ureterovesical or ureterovaginal junction using a 5-mm Hem-o-lok. After hemostasis was confirmed, two 5-mm or one 3-mm and one 5-mm skin incision was united, and the specimen was removed intact through the conjoined port sites, without a laparoscopic retrieval bag. After evacuation of the pneumoperitoneum and removal of the ports, without a drainage tube, the abdominal fascia was carefully closed with a 2-0 absorbable suture to prevent umbilical hernia development or wound dehiscence, and the transumbilical incision was conglutinated by tissue adhesive glue.

### Follow-up

The demographics, incision length, operative time, estimated blood loss, oral feeding time, length of stay, and perioperative complications for all patients were recorded and respectively analyzed. All patients were followed up for the symptoms, and the physical, urinary routine, ultrasonography, and/or renal scintigraphy examinations were performed at approximately 6, 12, 18, or 24 months postoperatively.

## Results

Of these cases, there were left-side in 12 patients, and right side in 4 patients. Their ages ranged from 7 to 62 months (mean: 33.8 months). TMLN was performed in all 16 cases, without additional ports or conversion to conventional laparoscopy or open surgery. The mean operative time was 108.4 min (range 90–125; Table 2). The estimated blood loss was almost nil, and no transfusion was required. Histopathological examination of all specimens revealed the renal dysplasia with or without inflammatory changes.

**Table 2:** **Intraoperative and post-operative details of patients**.

Characteristic	Transumbilical multiport laparoscopic nephroureterectomy
No. of cases	16
Incision length (cm)	1.3
Additional ports	0
Operative time (min)	108.4 ± 16.2 (range: 90–125)
Oral feeding (h)	36.3 ± 6.1 (32–48)
Return to normal activities (days)	2.8 ± 0.8 (2–4)
Hospital stay (days)	5.4 (range: 4–7)
Complication (%)
Wound infection	1 (6.3%)
Subcutaneous emphysema	1 (6.3%)
Internal organ damage	0 (0%)
Hematuria	0 (0%)
Urinary infection	0 (0%)

The post-operative recovery was uneventful in all patients. No patients needed analgesic use after operation for the tolerable levels of pain. The mean length of hospital stay was 5.4 days (Table 2), and oral fluid intake was resumed after 36 h of surgery (Table 2). All children returned to normal activity after 2–4 days of surgery (Table 2). There were no severe intraoperative or post-operative complications except for subcutaneous emphysema in one patient (Table 2), who recovered smoothly without any treatment. Another patient encountered slight wound infection, and recovered by administration of oral antibiotics (Table 2). All cases had an uneventful course after discharge. One month post-operation, the periumbilical wound was cosmetically satisfactory, without revealable scars within the belly button (Figure 1C).

All patients were followed up for 18–30 months (mean 22.6 months). No urinary incontinence occurred in all patients (Table 3). No umbilical hernias or urinary tract infection were found during the follow-up for all patients (Table 3). All patients had normal function of the contralateral kidney (Table 3).

**Table 3:** **Midterm follow-up results**.

Characteristic	Transumbilical multiport laparoscopic nephroureterectomy
No. of cases	16
Follow-up (months)	22.6 (range: 18–30)
Urinary incontinence	0
Umbilical hernia	0
Urinary infection	0
Contralateral kidney function
Normal	21
Abnormal	0

## Discussion

In 1991, Clayman et al. first described the laparoscopic nephrectomy (6). Since then, it has gained wide acceptance for the management of benign renal diseases in adults. In subsequent years, Erlich et al. (7) and Koyle et al. (8) initially reported the application of this technique in the pediatric population. Currently, laparoscopic nephrectomy or nephroureterectomy is generally used for benign renal conditions in pediatric patients, such as dysplastic kidneys and non-functional kidneys due to obstructive or refluxing uropathy (9). Both the transperitoneal and retroperitoneal approaches have been described for laparoscopic nephrectomy or nephroureterectomy. However, for pediatric surgeons familiar with the layout of abdominal cavity, the laparoscopic transperitoneal approach represents a natural progression from performing laparotomies (10).

Despite the widespread acceptance of standard multiple-port laparoscopic surgery, there have been efforts to further reduce its invasiveness and access-related complications. In recent years, LESS was established as general term for all the new surgical procedures using only one skin incision for access of camera and instruments (11). Specific access devices, such as TriPort, Quad-Port, Gelport, and Uni-X, are recently designed to allow multiple instruments to be passed through at the same time. However, the current single incision for LESS requires at least a 20-mm fascial incision, which is larger than the umbilicus in virtually all infants and small children (12). A recent study from Ham et al. used a homemade transumbilical port, and the length of umbilical incision was decreased from 2.0 to 1.0 cm once the surgeon gained experience (5). To perform LESS, some surgeons make either a semicircular or vertical umbilical incision, and insert multiple trocars through different fascial incisions after mobilizing the skin (13, 14). Because the LESS procedures need specific instruments to accomplish some degree of triangulation and make the surgery easier, such as a multichannel laparoscopic port, flexible laparoscopes or curved instruments, the high costs limit the wide utilization of LESS in many countries, especially those developing countries (15). In addition, the counterintuitive nature of this maneuver requires the surgeons’ retraining to master the technical skills, which may have steep learning curve and hamper the performance of true LESS in pediatric patients (16). Some authors have advocated using one straight instrument and one reticulating instrument through a single periumbilical wound to ease the dissection in one 10-year-old girl (13). Moreover, Tam et al. described the technique of crossing two straight instruments to widen both extra- and intracorporeal working spaces and facilitate instrumental maneuverability (14).

In our cases, we choose three separate skin incisions for trocar placement, with the aim to maximize spacing between the trocars within the limited umbilical wound. This may prevent the leakage of pneumoperitoneum, and the skin incisions are more cosmetic. The potential incision complications, such as incisional hernia and wound infection, are rare after this TMLN. In addition, we used conventional straight laparoscopic trocars and instruments to perform the procedures. Potential obstacles to single-port surgery include collision of instruments and hands, reduced intracorporeal work space, triangulation difficulties, and a steep learning curve. At the beginning, we indeed encountered difficulty in performing TMLN. Because the tight approximation of instrument placement restricts the surgeon’s hands to a narrow range of motion, it is technically changeling to accomplish the surgery in an efficient manner. The parallel alignment of these instruments also limits the triangulation, which is a founding principle of effective laparoscopic surgery. Moreover, in line placement of the telescope narrows the visual field, and forces the field of view to be limited by the movement of instruments. There are four main tricks to overcome these technical difficulties of TMLN. Firstly, three trocars are staggered at different heights to minimize collision at trocar heads. Secondly, two working trocars are lateral placed at 6 and 12 o’clock positions of the umbilical ring in order to maximize spacing between two working ports. These two trocars can be moderately and laterally placed along subcutaneous planes, resulting in adequate freedom of instrumental movement with reasonable triangulation and no torque on the trocars. The use of a 30° telescope provides better visualization for the manipulation of instruments. Thirdly, transabdominal hitch stitches can be introduced through the abdominal wall to lift the ureter or renal pelvis for easier dissection. The exact site of entry of the needle was determined under direct laparoscopic vision. Finally, the retrieval of laparoscopically resected specimens is difficult through the single working port. However, after conjoining the port sites, the specimen can be extracted easily. Once we ameliorate these techniques, we can accomplish the surgery in an efficient manner, which not only obviates the need for additional port site wounds, but also renders the operation virtually scarless.

Since the laparoscopic nephroureterectomy is a routine procedure in our center, we are prone to be familiar with the surgical steps of TMLN. Although there is still less range of motion, instrument crossing, and occasional instrument collision in TMLN, this procedure can be accomplished to achieve excellent results. Our intraoperative and post-operative data demonstrate that TMLN is a feasible and safe technique for renal dysplasia in pediatric patients. The average operative time in this series is <2 h, which compares favorably with that reported in standard laparoscopic nephroureterectomy (3). The learning curve may influence the operative time, but once we master this procedure, we can accomplish the surgery in an efficient and safe manner. Due to prolonged duration of preoperative examination and lack of specific instruments, treatment and experienced doctors in community hospitals and primary hospitals in our country, the hospital stay of patients was longer in our series than that in many developed countries (3). In our series, most patients did not suffer from post-operative pain, without the need of narcotic use. However, in the further work, we will measure the narcotic use or some objective pain scale in a larger series, especially when compared with open or traditional laparoscopic procedures.

## Conclusion

Transumbilical multiport laparoscopic nephroureterectomy is a feasible and safe technique for pediatric patients. It can be performed with usual laparoscopic instruments. Once the technical limitations are overcome, the experienced laparoscopic surgeons can accomplish the TMLN in an efficient manner. Since our study is retrospective, the true value of TMLN in outcome analysis may be affected by the inner limitations in small number of cases. The benefits and limitations of TMLN need to be further confirmed by a prospective randomized study with a large number of cases and a long-term follow-up. We believe that TMLN procedure is an alternative technique of LESS procedure, which may be useful for pediatric urologists to choose the surgical approaches for the management of renal dysplasia in children.

## Conflict of Interest Statement

The authors declare that the research was conducted in the absence of any commercial or financial relationships that could be construed as a potential conflict of interest.
